# Varietal Aromas of Fortified Wines from Different Moscato Var. (*Vitis vinifera* L.) under the Same Pedoclimatic Conditions

**DOI:** 10.3390/foods10112549

**Published:** 2021-10-22

**Authors:** Antonella Verzera, Maria Merlino, Fabrizio Cincotta, Ottavia Prestia, Antonio Sparacio, Salvatore Sparla, Concetta Condurso

**Affiliations:** 1 Department of Veterinary Sciences, University of Messina, Polo Universitario dell’Annunziata, Viale G. Palatucci, 98168 Messina, Italy; averzera@unime.it (A.V.); maria.merlino@unime.it (M.M.); ottavia.prestia@unime.it (O.P.); concetta.condurso@unime.it (C.C.); 2 Regional Institute of Wine and Oil, Via della Libertà 66, 90143 Palermo, Italy; antonio.sparacio@regione.sicilia.it (A.S.); salvatore.sparla@regione.sicilia.it (S.S.)

**Keywords:** Moscato varieties, fortified wines, varietal aromas, terpenes, HS-SPME-GC-MS

## Abstract

*Vitis vinifera* L. cv. Moscato includes different varieties mainly used to produce sweet wines, such as fortified wines. Moscato grapes are characterized by a large number of free and glycosylated monoterpenoids giving very aromatic wines. However, the literature data on the aroma profile of fortified Moscato wines are very limited. In light of this, the present research aimed to investigate the aroma compounds, mainly the varietal ones, of fortified wines from different Moscato varieties, namely Giallo (Yellow), Bianco (White), Bianco at Petit Grain (Blanc à Petits Grains), Ottonel and Rosa (Pink of Trentino), cultivated under the same pedoclimatic conditions. Using the HS-SPME-GC-MS (head space-solid phase micro extraction-gas chromatography-mass spectrometry) technique, numerous varietal and fermentative aroma compounds have been identified and quantified and significant differences were observed among varieties in the levels of mostly volatiles and in their ratios. Based on their composition, the studied wines can be divided in two groups depending on whether linalool or geraniol prevails among varietal aromas. These results are evidence that each Moscato variety has a typical varietal aroma composition, even if some similarities were found between the two white varieties, and between Moscato Giallo and Moscato Ottonel varieties. Moscato Rosa showed a peculiar aroma composition and the lowest ester/terpene ratio.

## 1. Introduction

Fortified wines, also known as liqueur or dessert wines, are characterized by an alcohol volume of between 15% and 22% [1]. They are produced by the addition of distilled spirits, during the wine making process, usually a neutral grape spirit, that provide microbial stability by creating an unfavorable environment for microbial action; it also causes chemical reactions such as oxidation, which lead to the fortified wines’ typical flavour and aroma profile, the desirable aged oxidative character that consumers expect from the product [2]. Fortified wines, historically originating in Europe, are currently produced worldwide, but Europe remains the key producer since most of these wines are based on European production procedures [3].

*Vitis vinifera* L. cv. Moscato includes different grape varieties which are characterized by a large number of free and glycosylated monoterpenoids [4]. Moscato varieties are, in fact, known to give aromatic wines and are mainly used for the production of sweet aromatic wines, namely Fortified, Sfursat and Passito Moscato. The grapes are mainly white with a medium-sized berry, spheroidal or slightly flattened in shape, yellow greenish colour which becomes golden yellow or amber when exposed to the sun. The grapevine, originating in the Mediterranean area, is one of the most ancient cultivated in the world. It has spread to many countries, mainly Italy and France, with different names, such as Moscato in Italy, Moscatel in America, Spain, and Portugal, Moscato a petits grains, Frontignan, Lunel or d’Alsace in France, Gelber muskateller in Germany and Tamioasa in Romania [5].

In Europe, Moscato Bianco (white Muscat) is the most cultivated variety, the oldest and most valuable; it is used for the production of the Italian Moscato d’Asti and for many French fortified wines known as vin doux naturels. Moscato Giallo (yellow Muscat) is widespread in Trentino Alto Adige (North-east region of Italy) with the name of Golden muskateller, where sparkling wines called Moscato Fior d’Arancio are also produced. Moscato di Alessandria (Muscat of Alexandria), also known as Zibibbo, is mainly cultivated in Pantelleria which is a Sicilian Island located in the middle of the Strait of Sicily (110 km SW from Sicily and 70 EN from Tunisia). Other varieties have been developed over the centuries, including Moscato Rosa (pink Muscat) mainly cultivated in Trentino Alto Adige where it is known as Rosen muskateller, Moscato Ottonel, and Moscato Petit-grain (Muscat blanc à Petits Grains) widespread in east Europe and limited to the north-west regions of Italy.

Despite increasing interest in sweet dessert wines, to the best of our knowledge, limited data have been reported in literature on the composition of fortified Moscato wines [6,7,8]. Moreover, little attention has been given to the aromatic volatile constituents determinant for the wine sensory features, and even less to the varietal aroma, such as mono and sesquiterpenes, except for some Portuguese Moscato varieties [7], and Moscato Nero d’Acqui (black Muscat) [8], an ancient aromatic Italian red grape variety sporadically found in old vineyards in the provinces of Asti and Alessandria (North Italy).

The knowledge of the terpenic profiles of the aromatic *Vitis vinifera* varieties, such as Moscato, is of great interest in enology and it is well known that terpene presence and concentration in grapes and wines depend, other than variety, on several factors, like cultivar, climate, soil, agricultural practices, phytotechnology and physiology of the vineyard, grape health status, and degree of ripeness of the grape [9,10,11].

As reported above, the aim of this work is to investigate the aroma compounds of fortified wines obtained from the most widespread Moscato varieties, cultivated under the same pedoclimatic conditions; a great attention will be given to the varietal aromas and mainly to the amount of terpenes considered key aroma compounds for Moscato wines.

## 2. Materials and Methods

### 2.1. Grape and Wine Samples

Grapes of *Vitis vinifera* L. cv. Moscato of the following varieties, Giallo (MG), Bianco (MB), Bianco at Petit Grain (MBPG), Ottonel (MO), and Rosa (MR) were cultivated in a experimental vineyard of the IRVO (Regional Institute of Wine and Oil, Palermo, Italy), located in Partinico (Palermo, Sicily, Italy, lat. 37°57′58.14′′, long. 13°03′37.67′′) at 350 m above the sea level. The vines were planted in 2013 with an inter-row spacing of 2.20 m and intra-row spacing of 0.90 m; the soil was of medium texture tending to sandy. The vineyard was vertical shoot-positioned with Guyot pruning and north–south oriented. The experiment was conducted over two different years, in 2018 and 2019. For the experimental design, randomized complete blocks (three blocks for each variety) were used. Harvest date was based on the technological maturity (about 20–21 Babo). For each variety, sugar content, pH, and total acidity were weekly assessed, during ripening until technological maturity, on 200 berries randomly collected from different positions.

After harvesting, both in 2018 and 2019, the grapes of each block were transferred to the IRVO experimental winery in Marsala (Sicily, Italy); for each variety, the grapes were divided into three parts which were subjected to the same treatments for wine production.

### 2.2. Wine Production

The grapes were hand harvested, destemmed, and crushed up to 0.6 bar of pressure with liquid yields of about 65%; the mash was then placed into a thermo-conditioned tank and added with tartaric acid to achieve a total acidity of not less than 6 g/L; thus, 10 g/hL of potassium metabisulphite (corresponding to 5 g/hL of SO_2_) were added. The must was cooled to 6–8 °C and clarified by static decanting in a stainless-steel tank to reach a turbidity of 100 NTU (nephelometric turbidity units). After about 6 h, 20 g/hL of *Saccharomyces cerevisiae* (ZYMAFLORE^®^ X5, Laffort, Bordeaux, France) and 8 g/hL of yeast nutrients (ammonium sulfate and ammonium phosphate) were added. Before addition, yeasts were rehydrated according to the protocol recommended by the manufacturer, paying attention to their acclimatization. Alcoholic fermentation was carried out at controlled temperature (16–18 °C). On the fermenting must, Babo, alcohol content, and fermentation temperature were monitored daily. When the residual sugar content in the fermenting must reached the level of about 100 g/L, the fermentation was stopped adding of 12 g/hL of potassium metabisulphite (corresponding to 6 g/hL of SO_2_) and 95% *v*/*v* food-grade ethanol up to a total alcohol content of about 15% *v*/*v*. The resulting wines were stored at 10 °C on the lees and batonnage was periodically applied, twice a week in the first month and once a week in the following months. After 3 months, racking, cold stabilization, clarification, and bottling were carried out. A total of 30 fortified wine samples, six for each Moscato variety, were obtained and each sample was analyzed in duplicate.

### 2.3. Chemical Analysis of Grapes and Wines

The chemical and physicochemical parameters in grapes and wines were determined according to the Council Regulation (EEC) No. 2676/90 Official Method [12]. Glycerol was determined by enzymatic assays [13].

### 2.4. Extraction of Volatile Aroma Compounds

The aroma volatiles were extracted applying the headspace solid phase microextraction technique in agreement with a previously optimized method [11,14,15]. In particular, a 40 mL vial equipped with a “mininert” valve (Supelco, Bellefonte, PA, USA) was filled with 20 mL of each wine sample. Extraction was performed in the headspace vial kept at 30 °C using a DVB/CAR/PDMS (divinylbenzene/carboxen/polydimethylsiloxane) fibre, of 50/30 μm film thickness (Supelco, Bellefonte, PA, USA). The sample was equilibrated for 15 min and then extracted for 20 min under continuous magnetic stirring. After sampling, the SPME fibre was kept for 3 min into the splitless injector of the GC/MS at 260 °C for the thermal desorption of the analytes onto the capillary GC column.

The GC analysis was performed using a Shimadzu GC 2010 Plus gas chromatograph directly interfaced with a TQMS 8040 triple quadrupole mass spectrometer (Shimadzu, Milan, Italy). The conditions were: VF-WAXms, 60 m, 0.25 mm i.d., 0.25 μm film thickness polar column (Agilent Technologies Italia S.p.A., Milan, Italy); oven temperature, 45 °C held for 5 min, then increased to 80 °C at a rate of 10 °C/min and to 240 °C at 2 °C/min; carrier gas, a constant flow of 1 mL/min; transfer line temperature, 250 °C; acquisition range, 40–200 m/z; scan speed, 1250 amu/s.

### 2.5. Identification and Quantification of Volatile Aroma Compounds

Each compound was identified using mass spectral data, NIST’18 (NIST/ EPA/NIH Mass Spectra Library, version 2.0, Gaithersburg, MD, USA), FFNSC 3.0 database, Linear Retention Indices (LRI), literature data and the injection of standards, where available, as reported by Cincotta et al. [16]. Selected ion monitoring (SIM) mode (SIM: *m*/*z* = 93.0 + 121.0 + 136.0) was used to visualize and identify terpenes in wine samples.

The volatile compounds have been quantified using the standard addition method, as previously reported [14]. Stock solutions of individual standards were prepared by dissolving the appropriate amount of each standard compound in ethyl alcohol (95%) to obtain a final concentration of 0.2 mg/mL. The solutions were stored at under −30 °C. Furthermore, five different amounts of each stock solution were added to multiple aliquots of each wine sample. The sample alone was also analysed. Quantification was based on a calibration curve generated by plotting detector response versus the amount spiked of each standard. The peak area of each compound was determined during three replicates, and the average value was calculated.

The standards used were purchased from Sigma Aldrich s.r.l. (Milan, Italy) at the highest purity available. To quantify compounds whose standards were not available, the calibration curve of a compound of the same chemical class with the most similar retention time was used (Table S1).

### 2.6. Statistical Analysis

Data were analyzed using XLStat software, version 2019.1.2 (Addinsoft, Damremont, Paris, France). One-way ANOVA (analysis of variance) and Duncan’s multiple range test at a confidence level of 95% were applied to physicochemical, chemical, and volatile data to determine significant differences among fortified wine samples from different Moscato varieties. Principal component analysis (PCA) and a clustering heatmap were also performed on volatile data in order to classify the fortified wines from different cultivars according to the varietal aromas.

## 3. Results

Table 1 reports the results of the physicochemical analyses carried out on the grapes at harvest and on the wine samples of the five varieties.

As regards grapes, statistically significant differences were observed only for the titratable acidity that was higher in MR samples (7.1 g/L). Also among wines, MR samples showed the highest values of titratable (6.3 g/L) and volatile (0.6 g/L) acidity. Residual sugars resulted statistically higher in MB, MO, and MR samples. The free and total SO_2_ was present in higher amount in MG and MBPG samples. All the other parameters did not show statistically significant differences among the fortified wines from different cultivars.

Seventy-one volatile compounds, pre-fermentative (C_6_ alcohols), fermentative (esters, higher alcohols, medium fatty acids) and varietal aromas (terpenes and C_13_-norisoprenoids), have been identified, most of them for the first time, in the wine samples of the different varieties. The identified pre-fermentative and fermentative, and varietal aroma compounds are reported in the supplementary materials (Tables S2 and S3), respectively, together with their LRI, odour, odour class, solubility in water and boiling point. The identified volatiles were quantified, and their amount is reported in Table 2 (pre-fermentative and fermentative aroma compounds) and Table 3 (varietal aroma compounds).

As regards esters, 30 volatiles were identified, mainly methyl and ethyl esters from C_2_ to C_14_, saturated and unsaturated, linear and branched. The esters are formed by yeasts during fermentation as secondary products and mainly contribute to wine aroma with positive fruity notes. In our samples the amount of the ester fraction showed quantitative significant differences among the sample wines, as well as the ratio among the main ester compounds. The highest content of esters was for MG wines, while MB and MBPG showed the lowest content. Ethyl acetate (OTV (odor threshold value), 7500 µg/L [17]), ethyl lactate (OTV, 54,000 µg/L [17]), and diethyl succinate (OTV, 300,000 µg/L [17]), with concentrations around mg/L, were the esters present at the highest levels in all the studied varieties but always inferior to their OTV. Ethyl octanoate, ethyl decanoate, and ethyl hexanoate were the most abundant among the remaining esters and have a very low odor threshold. Ethyl esters of short- and medium-chain-length carboxylic acids (C_2_–C_10_) and acetates of short-chain-length alcohols (C_4_–C_6_) are among the most important components of sensory perception of wines, since they are reported at the concentration above their OTV; however, at high concentrations, they can cover the varietal aromas, reducing the wine’s complexity [18].

Higher alcohols and aliphatic acids are also formed during wine fermentation. As regards the higher alcohols, which were the major constituents of the wine volatile fraction, isoamyl alcohol and β-phenylethyl alcohol showed the highest values in MO and MR wines. The opposite happened for the aliphatic acids which showed in these varieties the lowest amount. As regards aliphatic acids and higher alcohols, a limited influence on the wine aroma has been demonstrated probably due to their high OTV [19] and water solubility; they contribute to the general complexity of wine aroma, but they are not able to inhibit the perception of other volatile compounds [19].

Pre-fermentative aroma, namely C_6_ alcohols, have been identified and the highest amount occurred for MR samples. They arise from membrane lipids through the lipoxygenase pathway during technological operations prior to the fermentation, as well as during heating of must heating or grape maceration. Their concentration depends on grape variety, ripeness stage, treatments prior to fermentation, and temperature/duration of contact with the skins. At a low concentration, they contribute positively to the overall aroma of the wine [18].

**Figure 1:**
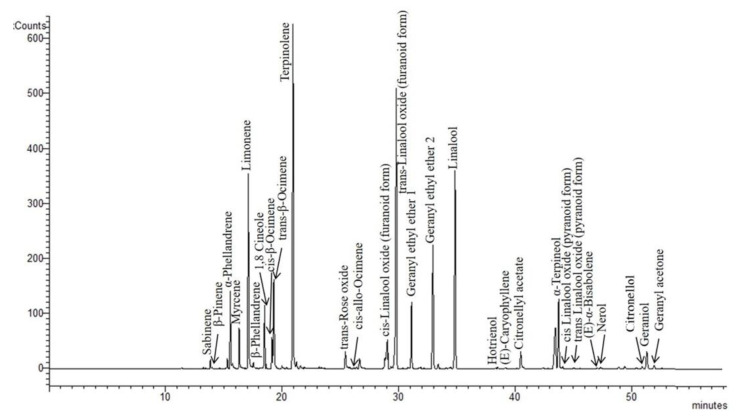
HS-SPME-GC-MS (head space-solid phase micro extraction-gas chromatography-mass spectrometry) profile in SIM (selected ion monitoring) mode (*m*/*z* = 93.0 + 121.0 + 136.0) of a fortified Moscato Giallo wine sample.

Although the volatile compounds generated in the fermentation process are usually the most important contributors to the overall aroma of the wine, the varietal aroma compounds originating in the vine play a fundamental role in the characteristics of many wines, among these, mainly Muscat wines. Indeed, terpenes, that are among the main varietal aroma contributors, have been detected in the highest amount in Moscato grapes; they can contain more than 5 mg/kg of terpenes, with linalool and geraniol the main ones [20]. Figure 1 reports a chromatogram of a Moscato wine sample in SIM mode (*m*/*z*: 93.00 + 121.00 + 136.00).

**Figure 2:**
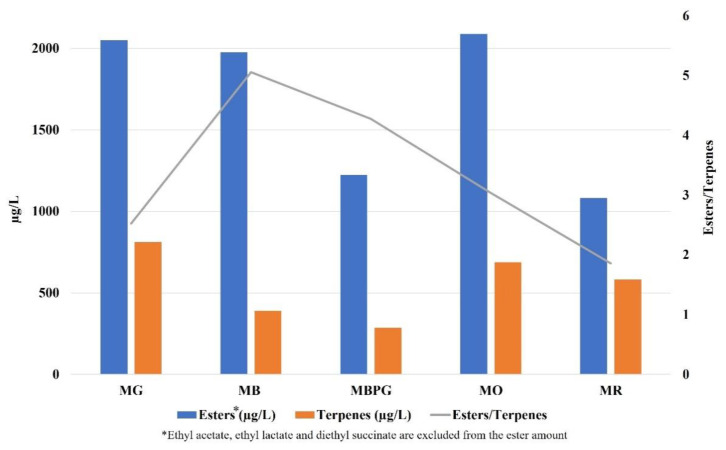
Amount of esters and terpenes, and their ratio in fortified Moscato wines from different varieties. MG = Moscato Giallo; MB = Moscato Bianco; MBPG = Moscato Bianco at Petit Grain; MO = Moscato Ottonel; MR = Moscato Rosa.

As shown in Figure 1, it is possible to verify the presence of a large number of terpenes, both hydrocarbons and oxygenated monoterpenes, and some were identified here for the first time. Considering the amount of each varietal aroma, a different composition resulted between the varieties not only for the amount of each volatile but also for the ratio among these. In all the varieties linalool, geraniol and trans-linalool oxide (furanoid form) were the main compounds among terpenes. Linalool had the highest concentration in MG, MO, and MR wines whereas geraniol was more prevalent in MB and MBPG wines. Our data are comparable only with those of Ossola et al. [8] who analyzed the amount of free and glycosylated monoterpenes in fortified Moscato nero d’Aqui wines using SPME-GC-MS; the total amount of terpenes is comparable with our results even if the amount of each terpene was different. In particular, Moscato Nero fortified wines have been characterized by a high amount of citronellol that prevailed among the other terpenes [8]. In our samples the total amount of terpenes ranged between 286 and 811.7 µg/L with the highest value in MG and MO wine samples (Figure 2). This content was higher than that reported by Ossola et al. in fortified wines [8].

Terpenes accumulate in the berry during ripening and are mainly responsible for the Moscato aroma [21]; the wines obtained from Moscato varieties are in fact defined as aromatic wines due to the high number of monoterpenes, both hydrocarbons and oxygenated [6].

Among C_13_-norisoprenoids, 6-methyl-5-hepten-2-one and geranyl acetone have been identified; these substances arise from the carotenoid degradation. The highest amount of these compounds was found in MR wines.

The ratio among the esters (fruity notes) and the terpenes (floral notes) is reported in Figure 2 showing interesting differences among varieties: the amount of esters was 2.5–3 times higher than terpenes in yellow varieties (MG and MO), 4.3–5-times higher in white varieties (MB and MBPG), less than 2 times in MR.

**Figure 3:**
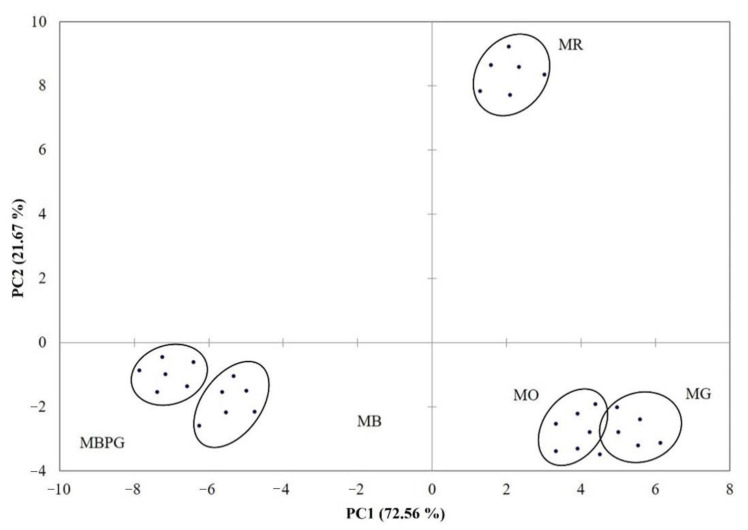
Principal component analysis (PCA) plot showing the multivariate variation among the fortified Moscato wines from different varieties in terms of volatile aroma compounds. MG = Moscato Giallo; MB = Moscato Bianco; MBPG = Moscato Bianco at Petit Grain; MO = Moscato Ottonel; MR = Moscato Rosa.

The PCA (Figure 3), performed on all volatile aroma compounds of fortified Moscato wines, showed a clear separation among samples: the wine samples were separate along PC1 (72.56% of the total variance) according to their berry color with MB and MBPG samples on the negative side and MR, MG, and MO on the positive one. MR wine samples were further separated from the others along PC2 (21.67% of the total variance), standing alone on the positive side of both PCs. This suggest that the volatile profile of fortified Moscato wines is influenced by the grape variety with particular regard to the berry color.

**Figure 4:**
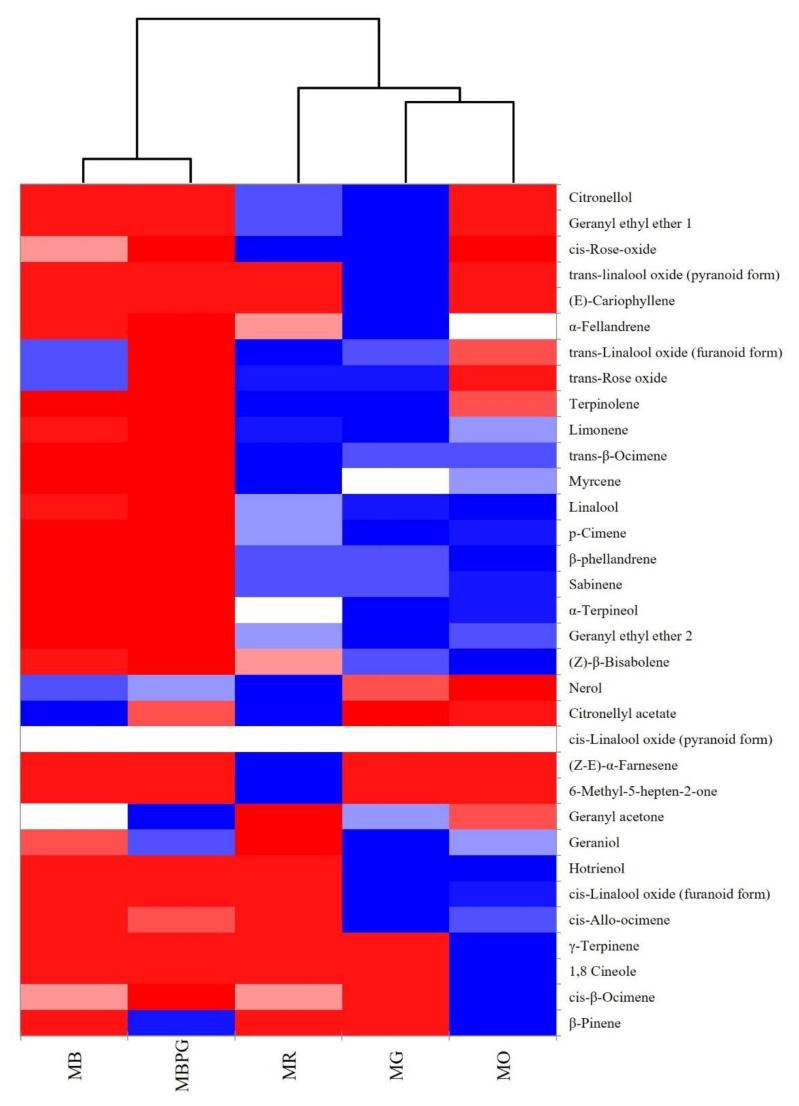
Heat map of the fortified Moscato wines from different varieties in terms of varietal aroma compounds.

Figure 4 reports the heat maps obtained by using the amount of varietal aromas of the fortified Moscato wines. The heatmap colors of the matrix indicate the strength of the correlation between the samples and varietal aroma compounds: red indicates a positive correlation (+1); dark blue indicates a negative correlation (−1); finally, shade indicates an in-between correlation. Two main big clusters are present: one including MB and MBPG, resulting in being more similar for varietal aroma composition, and another one including two sub clusters, the first with MG and MO samples and the second with MR samples. These results evidenced that each Moscato variety has a typical varietal aroma composition, even if similarities were observed between the two white varieties, MB and MBPG, and between MG and MO varieties whereas MR had a peculiar composition in agreement with the PCA results reported above. The peculiarity of MR fortified Moscato wines emerged also from a cellar tasting (data not reported), that highlighted a more delicate and floral aroma of this wine than others, probably due to the different ratio between ester and terpene amounts.

## 4. Conclusions

The results of the present study allowed us to characterize the volatile aroma profile of fortified Moscato wines from different varieties. A large number of varietal aromas, mainly monoterpenes and sesquiterpenes, hydrocarbons and their oxygenated derivatives, have been identified and quantified. Since the analyzed wines have been obtained from grapes cultivated under the same pedoclimatic conditions, the different in varietal aromas can be related to the Moscato variety. The volatile aroma fraction of all the varieties is characterized by a high amount of terpenes, even if a different ratio among each other as well as among fermentative aromas resulted. Among the studied varieties, Moscato Rosa cv stands out above all for the peculiarity of its aroma. The increase of knowledge about the wine varietal aroma compounds could help enologists to obtain high-quality Moscato dessert wines.

## Figures and Tables

**Table 1:** Physicochemical parameters (average values) of grapes and wines from different Moscato cv.

	MG ^1^	MB ^1^	MBPG ^1^	MO ^1^	MR ^1^
Grapes					
Babo	20.10	21.50	21.00	21.00	21.10
Titratable Acidity (g/L)	6.2 ^b,2^	5.9 ^b^	5.6 ^b^	6.0 ^b^	7.1 ^a^
pH	3.28	3.30	3.38	3.29	3.29
Wines					
Alcohol %vol.	15.40	15.70	15.33	15.50	15.30
Gross extract (g/L)	82.8 ^c^	113.0 ^a^	95.1 ^b^	107.5 ^b^	122.3 ^a^
pH	3.60	3.31	3.50	3.16	3.40
Titratable Acidity (g/L)	4.9 ^b^	5.2 ^b^	5.4 ^b^	6.2 ^a^	6.3 ^a^
Volatile Acidity (g/L)	0.50 ^b^	0.48 ^b^	0.40 ^b^	0.36 ^b^	0.60 ^a^
Free SO_2_ (mg/L)	14.0	14.0	18.0	13.0	17.0
Total SO_2_ (mg/L)	127.0 ^a^	83.0 ^b^	119.5 ^a^	81.0 ^b^	65.5 ^b^
Malic Acid (g/L)	1.40	1.19	1.00	1.43	1.30
Residual Sugars (g/L)	60.3 ^b^	81.3 ^a^	62.9 ^b^	75.8 ^a^	87.6 ^a^
Glycerol (g/L)	6.6	7.0	7.5	6.7	7.4

^1^ Grapewine varieties: MG = Moscato Giallo; MB = Moscato Bianco; MBPG = Moscato Bianco at Petit Grain; MO = Moscato Ottonel; MR = Moscato Rosa. ^2^ Different uppercase letters in the same row represent significant differences at *p* < 0.05 by Duncan’s multiple range test.

**Table 2:** Pre-fermentative and fermentative aroma compounds quantified (average values ^1^) in fortified wines from different Moscato cv.

Compounds	MG ^2^	MB ^2^	MBPG ^2^	MO ^2^	MR ^2^
Esters (µg/L)					
Ethyl acetate ^3^	7.948 ^a,4^	2.043 ^b^	1.339 ^c^	7.244 ^a^	2.720 ^b^
Ethyl butanoate	2.47 ^a^	1.16 ^b^	0.78 ^b^	2.13 ^a^	1.00 ^b^
Ethyl 2-methyl-butanoate	0.26	0.54	0.43	0.54	0.21
Ethyl 3-methyl-butanoate	0.47	0.49	0.46	0.92	0.40
Isoamyl acetate	20.47 ^b^	30.20 ^a^	21.42 ^b^	18.94 ^b^	16.07 ^b^
Ethyl hexanoate	105.50 ^a^	108.18 ^a^	65.64 ^b^	109.10 ^a^	59.15 ^b^
Hexyl acetate	3.26 ^c^	17.02 ^a^	9.07 ^b^	2.81 ^c^	1.42 ^c^
3-Hexenyl acetate	0.46 ^a^	0.39 ^a^	0.64 ^a^	0.05 ^b^	0.40 ^a^
Ethyl heptanoate	1.29	1.35	1.13	1.16	2.91
Ethyl lactate ^2^	0.710 ^a^	0.390 ^b^	0.220 ^b^	0.810 ^a^	0.781 ^a^
Methyl octanoate	1.03	1.05	0.70	1.05	0.75
Ethyl octanoate	1056.83 ^a^	942.13 ^a^	586.38 ^b^	1251.96 ^a^	502.53 ^b^
Isoamyl hexanoate	1.65 ^b^	1.30 ^b^	0.96 ^b^	4.48 ^a^	1.43 ^b^
(*E*)-4-Ethyl octenoate	0.93	0.92	1.10	0.68	0.61
Propyl octanoate	0.95	0.51	0.48	0.88	0.46
Ethyl nonanoate	2.53 ^a^	2.15 ^a^	1.19 ^b^	0.78 ^b^	1.28 ^b^
Butyl octanoate	1.09 ^b^	2.68 ^a^	1.68 ^a^	0.63 ^b^	2.28 ^a^
Methyl decanoate	0.80	0.94	0.74	0.66	1.43
Ethyl decanoate	637.73 ^a^	651.87 ^a^	379.39 ^b^	557.79 ^a^	308.79 ^b^
Isoamyl octanoate	4.79	3.86	2.99	5.06	2.48
Diethyl succinate ^2^	4.960 ^b^	4.460 ^b^	3.450 ^b^	4.380 ^b^	7.480 ^a^
Ethyl (*E*)-4-decenoate	1.50 ^b^	1.63 ^b^	0.80 ^c^	0.42 ^c^	2.44 ^a^
Ethyl (*Z*)-4-decenoate	159.63 ^a^	159.77 ^a^	119.62 ^a^	86.29 ^b^	144.50 ^a^
Ethyl (*E*)-3-decenoate	3.73 ^a^	3.99 ^a^	1.75 ^b^	1.76 ^b^	0.53 ^c^
Ethyl (*Z*)-3-decenoate	3.98 ^a^	1.86 ^b^	0.79 ^c^	0.86 ^c^	0.46 ^c^
Isobutyl decanoate	0.32	0.47	0.31	0.26	0.23
Methyl dodecanoate	0.29	0.28	0.22	0.26	0.41
β-Phenyl-ethyl acetate	4.95 ^a^	4.45 ^a^	2.39 ^b^	4.77 ^a^	2.98 ^b^
Ethyl dodecanoate	29.37 ^a^	36.50 ^a^	19.77 ^b^	32.80 ^a^	24.47 ^b^
Isoamyl decanoate	1.32	0.99	0.90	1.13	0.88
Ethyl tetradecanoate	2.26 ^a^	0.45 ^c^	0.84 ^b^	1.14 ^b^	0.80 ^b^
Alcohols (mg/L)					
Isoamyl alcohol	30.06 ^b^	28.64 ^c^	23.59 ^c^	41.90 ^a^	45.66 ^a^
1-Hexanol	1.45 ^b^	0.88 ^b^	1.15 ^b^	1.42 ^b^	3.11 ^a^
(*Z*)-3-Hexen-1-ol	0.03 ^b^	- ^5,c^	- ^c^	0.03 ^b^	0.15 ^a^
β-phenyl-ethyl alcohol	10.18 ^a^	5.16 ^b^	4.36 ^b^	14.72 ^a^	15.84 ^a^
Acids (mg/L)					
Octanoic acid	2.36 ^a^	2.71 ^a^	2.65 ^a^	1.07 ^b^	0.49 ^c^
Decanoic acid	0.96 ^a^	0.48 ^b^	0.58 ^b^	0.11 ^c^	0.47 ^b^
Others (µg/L)					
4-Methyl tiazole	0.031	0.021	0.010	0.04	0.050

^1^*n* = 12, six samples in duplicate. ^2^ Grapewine varieties: MG = Moscato Giallo; MB = Moscato Bianco; MBPG = Moscato Bianco at Petit Grain; MO = Moscato Ottonel; MR = Moscato Rosa. ^3^ Amount reported in mg/L. ^4^ Different uppercase letters in the same row represent significant differences at *p* < 0.05 by Duncan’s multiple range test. ^5^ Not quantified.

**Table 3:** Varietal aroma compounds quantified (average values ^1^) in fortified wines from different Moscato cv.

Compounds	MG ^2^	MB ^2^	MBPG ^2^	MO ^2^	MR ^2^
Terpenes (µg/L)					
Hydrocarbons Monoterpenes					
Sabinene	1.00 ^a,3^	- ^4,b^	- ^b^	1.12 ^a^	1.00 ^a^
β-Pinene	- ^b^	- ^b^	0.76 ^a^	1.06 ^a^	- ^b^
α-Phellandrene	10.90 ^a^	1.67 ^c^	0.62 ^c^	4.48 ^b^	3.72 ^b^
Myrcene	2.06 ^b^	0.15 ^c^	0.08 ^c^	2.13 ^b^	4.08 ^a^
Limonene	33.30 ^a^	8.19 ^c^	3.48 ^c^	22.71 ^b^	30.50 ^a^
β-Phellandrene	1.00 ^a^	- ^b^	- ^b^	1.23 ^a^	1.00 ^a^
*cis*-β-Ocimene	3.60 ^b^	5.86 ^b^	0.06 ^c^	16.33 ^a^	5.73 ^b^
*trans*-β-Ocimene	11.00 ^a^	- ^b^	- ^b^	11.45 ^a^	16.00 ^a^
γ-Terpinene	- ^b^	- ^b^	- ^b^	0.19 ^a^	- ^b^
*p*-Cimene	28.94 ^a^	5.81 ^b^	2.91 ^b^	28.34 ^a^	21.15 ^a^
Terpinolene	9.00 ^a^	- ^c^	- ^c^	3.13 ^b^	9.00 ^a^
*cis*-Allo-ocimene	1.00 ^a^	- ^b^	0.20 ^a^	0.65 ^a^	- ^b^
Oxygenated Monoterpenes					
1,8 Cineole	- ^b^	- ^b^	- ^b^	1.34 ^a^	- ^b^
*cis*-Rose-oxide	0.82 ^a^	0.39 ^b^	0.04 ^c^	0.10 ^c^	0.74 ^a^
*trans*-Rose oxide	2.79 ^a^	2.49 ^a^	0.52 ^c^	1.41 ^b^	2.88 ^a^
*cis*-Linalool oxide (furanoid form)	9.00 ^a^	- ^b^	- ^b^	6.34 ^a^	- ^b^
*trans*-Linalool oxide (furanoid form)	88.49 ^a^	88.18 ^a^	91.58 ^a^	58.74 ^b^	103.18 ^a^
Geranyl ethyl ether 1	16.88 ^a^	1.57 ^b^	1.25 ^b^	0.76 ^c^	10.74 ^a^
Geranyl ethyl ether 2	46.43 ^a^	5.62 ^c^	4.24 ^c^	31.26 ^b^	28.01 ^b^
Linalool	240.44 ^a^	109.48 ^b^	55.57 ^b^	255.08 ^a^	204.53 ^a^
Hotrienol	3.23 ^a^	0.84 ^c^	0.55 ^c^	2.85 ^b^	0.37 ^c^
Citronellyl acetate	- ^d^	1.91 ^b^	0.79 ^c^	0.68 ^c^	2.13 ^a^
α-Terpineol	73.35 ^a^	0.98 ^c^	3.19 ^c^	60.43 ^a^	41.64 ^b^
*cis*-Linalool oxide (pyranoid form)	-	-	-	-	-
*trans*-linalool oxide (pyranoid form)	2.00 ^a^	- ^b^	- ^b^	- ^b^	- ^b^
Nerol	13.04 ^a^	17.42 ^a^	15.60 ^a^	7.46 ^b^	19.16 ^a^
Citronellol	2.04 ^a^	0.33 ^b^	0.25 ^b^	0.05 ^c^	1.17 ^a^
Geraniol	207.96 ^a^	138.67 ^a^	185.45 ^a^	165.60 ^a^	72.14 ^b^
C_13_-Norisoprenoids					
6-Methyl-5-hepten-2-one	- ^c^	- ^c^	- ^c^	- ^c^	0.65 ^a^
Geranyl acetone	0.75	0.71	0.89	0.60	0.42
Sesquiterpenes					
(*E*)-Cariophyllene	0.70 ^a^	- ^b^	- ^b^	- ^b^	- ^b^
(*Z*-*E*)-α-Farnesene	- ^b^	- ^b^	- ^b^	- ^b^	1.06 ^a^
(*Z*)-β-Bisabolene	2.00 ^a^	0.64 ^b^	- ^c^	2.78 ^a^	1.08 ^b^

^1^ *n* = 12, six samples in duplicate. ^2^ Grapewine varieties: MG = Moscato Giallo; MB = Moscato Bianco; MBPG = Moscato Bianco at Petit Grain; MO = Moscato Ottonel; MR = Moscato Rosa. ^3^ Different uppercase letters in the same row represent significant differences at *p* < 0.05 by Duncan’s multiple range test. ^4^ Not quantified.

## Data Availability

The data presented in this study are available on request from the corresponding author.

## Supplementary Materials

The following are available online at https://www.mdpi.com/article/10.3390/foods10112549/s1, Table S1: Quality parameter of the optimized HS-SPME-GC-MS method, Table S2: Pre-fermentative and fermentative aroma compound identified in Moscato fortified wines, Table S3: Varietal aroma compounds identified in Moscato fortified wines.
