# Supplementary material for: Varietal Aromas of Fortified Wines from Different Moscato Var. (Vitis vinifera L.) under the Same Pedoclimatic Conditions
Source: Foods. 2021 Oct 22;10(11):2549. doi: 10.3390/foods10112549 (PMC8622996; doi:10.3390/foods10112549)
Supplement: Supplementary file 1 [file foods-10-02549-s001.zip › foods-1405445-supplementray.pdf]

Table S1. Quality parameter of the optimized HS-SPME-GC–MS method.

Compound	Linear range (μg/L)	Slope	R^2	Compounds not available and quantified whit the same calibration curve
**Esters**				
Ethyl acetate[1]	0.100-10.000	2.648	0.9994	-
Ethyl 3-methyl-butanoate	0.10-10.00	1.345	0.9996	Ethyl butanoate, Ethyl 2-methyl-butanoate
Isoamyl acetate	10.00-50.00	3.104	0.9989	-
Ethyl hexanoate	10.00-200.00	2.531	0.9991	-
Hexyl acetate	0.50-50.00	2.860	0.9993	-
Ethyl lactate[1]	0.100-1.000	1.935	0.9987	-
Methyl octanoate	0.01-5.00	1.673	0.9994	3-Hexenyl acetate, Ethyl heptanoate, Isoamyl hexanoate, Ethyl (*E*)-4-octenoate
Ethyl octanoate	100.00-2000.00	2.482	0.9984	Ethyl decanoate
Ethyl nonanoate	0.10-10.00	3.067	0.9999	Propyl octanoate, Methyl decanoate
Isoamyl octanoate	0.50-10.00	2.751	0.9989	Butyl octanoate
Diethyl succinate[1]	1.000-10.000	2.105	0.9991	-
Ethyl (*E*)-4-decenoate	0.20-200.00	3.781	0.9995	Ethyl (*Z*)-4-decenoate, Ethyl (*E*)-3-decenoate, Ethyl (*Z*)-3-decenoate
β-Phenyl-ethyl acetate	1.00-10.00	1.892	0.9986	-
Ethyl dodecanoate	10.00-100.00	3.244	0.9993	-
Isoamyl decanoate	0.10-2.00	2.984	0.9995	Isobutyl decanoate, Methyl dodecanoate
Ethyl tetradecanoate	0.20-20.00	3.137	0.9993	
**Alcohols**				
Isoamyl alcohol[1]	10.00-100.00	2.309	0.9983	-
1-Hexanol[1]	0.10-10.00	1.894	0.9992	-
(*Z*)-3-Hexen-1-ol[1]	0.01-1.00	1.093	0.9995	-
β-phenyl-ethyl alcohol[1]	1.00-20.00	2.845	0.9985	-
**Acids**				-
Octanoic acid[1]	0.10-5.00	3.084	0.9983	Decanoic acid
**Others**				
4-Methyl tiazole	0.010-0.100	1.059	0.9992	-
**Hydrocarbons**				
**Monoterpenes**				
β-Pinene	0.20-10.00	2.845	0.9997	Sabinene
Limonene	0.50-50.00	1.982	0.9993	α-Phellandrene, β-phellandrene, γ-Terpinene, *p*-Cimene, Terpinolene
cis-β-Ocimene	0.05-50.00	2.672	0.9994	Myrcene, *trans*-β-Ocimene, *cis*-Allo-ocimene

**Oxygenated Monoterpenes**				
1,8 Cineole	0.05-5.00	1.894	0.9989	-
trans-Rose oxide	0.02-200.00	2.275	0.9982	*cis*-Rose-oxide, *cis*-Linalool oxide (furanoid form), *trans*-Linalool oxide (furanoid form), *cis*-Linalool oxide, (pyranoid form), *trans*-linalool oxide (pyranoid form)
Geranyl ethyl ether 2	0.50-50.00	3.132	0.9991	Geranyl ethyl ether 1
Linalool	0.05-500.00	1.084	0.9993	Hotrienol
Citronellyl acetate	0.10-10.00	2.084	0.9995	-
α-Terpineol	0.10-100.00	1.927	0.9992	-
Nerol	0.01-50.00	2.957	0.9986	Citronellol
Geraniol	0.50-500.00	1.474	0.9993	-
**C_{13}-Norisoprenoids**				
6-Methyl-5-hepten-2-one	0.05-5.00	3.044	0.9994	-
Geranyl acetone	0.05-5.00	2.654	0.9993	-
**Sesquiterpenes**				
(*E*)-Caryophyllene	0.10-10.00	3.054	0.9982	-
(*Z*-*E*)-α-Farnesene	0.10-5.00	2.994	0.9995	(*Z*)-β-Bisabolene

[1] Linear range value expressed in mg/L.

Table S2. Pre-fermentative and fermentative aroma compound identified in Moscato fortified wines.

Compounds	LRI	Odour[1]	Main odour class*	Solubility in water mg/L @ 25 °C	Boiling point °C @ 760.00 mm Hg
**Esters**					
Ethyl acetate	839	ethereal, fruity, sweet, grape, rummy	ethereal	8.00E+4	76.50
Ethyl butanoate	1035	sweet, fruity	fruity	4900	120
Ethyl 2-methyl-butanoate	1051	fruity, berry	fruity	1070	132
Ethyl 3-methyl-butanoate	1065	sweet, fruity, pineapple, apple	fruity	2000	131
Isoamyl acetate	1115	sweet, banana, fruity	fruity	2000	142
Ethyl hexanoate	1226	sweet, fruity, pineapple, waxy	fruity	629	166
Hexyl acetate	1265	green, fruity, sweet, fatty	fruity	511	170
3-Hexenyl acetate	1320	fruity, green, banana, pear	fruity	480.5	174
Ethyl heptanoate	1326	fruity, pineapple, sweet	fruity	290	188
Ethyl lactate	1341	sweet, fruity, acidic, etherial	fruity	1.00E+6	154
Methyl octanoate	1384	waxy, green, sweet, orange	waxy	64	194
Ethyl octanoate	1435	waxy, sweet, musty, pineapple	waxy	70.1	206
Isoamyl hexanoate	1452	fruity, sweet, pineapple	fruity	12.56	225
Ethyl (*E*)-4-octenoate	1480	fruity, pear, citrus	fruity	52.1	-
Propyl octanoate	1513	coconut, cocoa, cognac	coconut	10.87	225
Ethyl nonanoate	1529	waxy, soapy, cognac	waxy	10.87	228
Butyl octanoate	1547	butter, ether, herbal	buttery	3.517	240
Methyl decanoate	1588	oily, wine fruity, floral	fermented	4.41	224
Ethyl decanoate	1637	sweet, waxy, fruity, apple	waxy	3.517	241
Isoamyl octanoate	1652	sweet, oily fruity, green	fruity	1.309	267
Diethyl succinate	1669	mild fruity, cooked apple	fruity	5547	217
Ethyl (*E*)-4-decenoate	1686	green, fruity, oily	green	5.496	-
Ethyl (Z)-4-decenoate	1699	-	-	5.496	236
Ethyl (E)-3-decenoate	1696	-	-	-	-
Ethyl (Z)-3-decenoate	1712	-	-	-	-
Isobutyl decanoate	1750	oily, sweet, brandy, apricot	fermented	0.4196	265
Methyl dodecanoate	1789	waxy, soapy, creamy, coconut	waxy	0.8841	261
β-Phenyl-ethyl acetate	1799	sweet, honey, floral rosy	floral	710.8	238
Ethyl dodecanoate	1826	sweet, waxy, soapy	waxy	0.4128	269
Isoamyl decanoate	1846	waxy, banana, fruity	waxy	0.134	286
Ethyl tetradecanoate	2038	sweet, waxy	waxy	0.03693	-
**Alcohols**					
Isoamyl alcohol	1207	fusel, alcoholic, pungent	fermented	2.67E+4	131
1-Hexanol	1345	pungent, ethereal, fusel oil	herbal	6885	156
(Z)-3-Hexen-1-ol	1374	green, grassy, melon	green	1.6E+4	156.50
β-phenyl-ethyl alcohol	1903	sweet, floral, fresh, rosey	floral	2.199E+4	219
**Acids**					
Octanoic acid	2043	fatty, waxy, rancid, oily, vegetable, cheesy	fatty	789	237
Decanoic acid	2267	rancid, sour, fatty, citrus	fatty	47.89	268
**Others**					
4-Methyl tiazole	1312	alliaceous, ripe, nutty, vegetable	nutty	1.746E+4	133

[1] Odour and main odour class are reported from the http://www.thegoodscentscompany.com.

[2] Odour Threshold Value.

Table S3. Varietal aroma compounds identified in Moscato fortified wines.

Compounds	LRI	Odour[1]	Main odour class*	Solubility in water mg/L @ 25 °C	Boiling point °C @ 760.00 mm Hg
**Terpenes**					
**Hydrocarbons Monoterpenes**					
Sabinene	1110	woody, spicy, citrus	woody	2.494	163
β-Pinene	1122	woody, piney and turpentine-like	herbal	7.061	163
α-Phellandrene	1167	citrus, terpenic, slightly green	terpenic	2.862	175
Myrcene	1170	herbaceous, woody	spicy	6.923	166
Limonene	1190	sweet, citrus and peely	citrus	13.8	175
β-Phellandrene	1209	mint, terpentine	minty	2.452	171
cis-β-Ocimene	1243	floral, herb, flower, sweet	floral	2.012	177
trans-β-Ocimene	1250	warm, floral, sweet	floral	6.923	174
γ-Terpinene	1252	terpy, sweet, citrus	terpenic	8.68	181
p-Cimene	1277	chemical, woody, terpenic, citrus	terpenic	27.88	176
Terpinolene	1288	sweet, fresh, piney, citrus	herbal	9.5	183
cis-Allo-ocimene	1370	sweet, floral, nut, skin, peppery	floral	2.348	188
**Oxygenated Monoterpenes**					
1,8 Cineole	1211	eucalyptus, herbal, camphor, medicinal	herbal	332.1	176
cis-Rose-oxide	1350	green, vegetative, floral, herbal	floral	764	-
trans-Rose oxide	1368	green, vegetative, floral, herbal	floral	764	196
cis-Linalool oxide (furanoid form)	1427	earthy, floral, sweet, woody	floral	1669	188
trans-Linalool oxide (furanoid form)	1446	earthy, floral, sweet, woody	floral	1669	188
Geranyl ethyl ether 1	1471	ethereal, fruity, green	fruity	5.618	218
Geranyl ethyl ether 2	1504	-	-	-	-
Linalool	1537	citrus, orange, floral, waxy, rose	floral	1590	194
Hotrienol	1601	sweet tropical ocimene fennel ginger	tropical	406.5	229
Citronellyl acetate	1654	floral, rosy, green, fatty	floral	5.686	229
α-Terpineol	1690	pine-like, woody	terpenic	710	214
cis-Linalool oxide (pyranoid form)	1699	citrus green	citrus	3992	201
trans-linalool oxide (pyranoid form)	1707	woody	woody	1669	188
Nerol	1745	fresh, citrus, floral, green	floral	255.8	225
Citronellol	1751	floral, rosey, waxy, herbal	floral	18.24	240
Geraniol	1814	floral, sweet, rosey, fruity	floral	100	229
**C_{13}-Norisoprenoids**					
6-Methyl-5-hepten-2-one	1336	fruity, apple, musty	citrus	3351	173.10
Geranyl acetone	1840	fresh, rose, leaf, floral, green	floral	8.867	247
**Sesquiterpenes**					
(*E*)-Caryophyllene	1615	spicy, woody and terpenic	spicy	0.05011	256
(*Z*-*E*)-α-Farnesene	1729	woody, green, vegetative	woody	0.01053	260
(*Z*)-β-Bisabolene	1739	balsamic, woody, spicy	balsamic	0.009945	274

[1] Odour and main odour class are reported from the http://www.thegoodscentscompany.com.

[2] Odour Threshold Value.
